# Training and credentialing in robotic general surgery

**DOI:** 10.1007/s00384-026-05129-3

**Published:** 2026-05-11

**Authors:** Matthew Harris, Helen Mohan, Bruno Augusto Alves Martins, Rahila Essani, Avanish Saklani, Deena Harji

**Affiliations:** 1 Division of Cancer Sciences, School of Medical Sciences, Faculty of Biology, Medicine and Health, University of Manchester, Manchester, UK; 2 Department of Surgery, Peter MacCallum Cancer Centre, Melbourne, VIC Australia; 3 Department of Colorectal Surgery, Hospital Universitário de Brasília, Brasília, Brazil; 4 Department of Colorectal Surgery, AdventHealth, Orlando, FL USA; 5 Tata Memorial Hospital, Mumbai, India; 6 Department of Colorectal Surgery, Manchester University NHS Foundation Trust, Manchester, UK

**Keywords:** Robotic, Training, Credentialing, Simulation

## Abstract

**Introduction:**

Robotic-assisted surgery is now embedded across many domains of general surgery; however, training and credentialing frameworks have not evolved at the same pace as technological adoption. As a result, significant variability exists in access to training, definitions of competency and proficiency, assessment standards, and institutional credentialing practices. These inconsistencies raise concerns regarding patient safety, equity of access, and workforce preparedness, particularly in the context of expanding robotic platforms and parallel learning curves among trainees and established surgeons.

**Methods:**

This narrative review aims to (1) clarify and define key concepts relevant to robotic surgical training, including competency, proficiency, benchmarking, and credentialing; (2) synthesise current barriers to effective robotic training across the surgical career continuum; and (3) propose a structured, standardised, and platform-agnostic credentialing pathway for robotic general surgery. Drawing on published literature and international consensus work, we identify challenges related to system access, lack of standardisation, training capacity, service pressures, and limited use of objective performance metrics and feedback mechanisms.

**Results:**

We propose a competency-based credentialing framework incorporating simulation-based foundational training, modular procedural progression, non-technical skills development, structured mentorship, and objective assessment using validated metrics. The pathway is designed to be adaptable across institutions and applicable to both surgical trainees and consultants, while remaining independent of vendor-specific credentialing models.

**Conclusion:**

Establishing a standardised approach to robotic training and credentialing is essential to ensure safe implementation, support proficiency development, and enable equitable access to robotic surgery. Coordinated action from professional bodies, training institutions, and healthcare systems will be required to deliver a future-ready robotic surgical workforce.

## Introduction

The use of robotic-assisted surgery has increased dramatically over the past two decades, transforming operative practice across multiple surgical specialities. In the speciality of general surgery, this evolution is particularly evident in procedures such as colorectal resections [1], hernia repairs [2], and foregut operations [3, 4], where robotic systems offer improved dexterity, three-dimensional visualisation, and enhanced ergonomics compared to traditional laparoscopic approaches [5]. These advances have contributed to better outcomes in selected patient populations, including reduced length of stay and lower conversion rates to open surgery [6].

Existing surgical robots in general surgery are telemanipulator systems, consisting of a surgeon-controlled console, robotic arms equipped with miniaturised instruments, and a high-definition vision system. The most widely used platform is the Da Vinci Surgical System; however, the landscape is rapidly diversifying with the introduction of alternative platforms, each with unique user interfaces and operating characteristics. Despite the proliferation of robotic technology, the training pathways for general surgeons, both in training and in independent practice, remain highly variable, inconsistently regulated and driven by industry stakeholders [7]. As the number of robotic surgical systems increases, it is imperative to ensure training standards are robust, replicable, and standardised across all available and emerging platforms. This narrative review explores key definitions, barriers, and solutions to robotic training and proposes a standardised pathway to credentialing.

Traditional surgical training models are not readily applicable to robotic surgery, with a lack of consideration of the unique learning curve associated with robotic surgery, with regard to the acquisition of platform, procedural and operative skills. Furthermore, current definitions of key concepts such as competency, proficiency, and credentialing are inconsistently applied [8, 9]. Proposed definitions in the context of robotic surgery are outlined in Table 1. Competency refers to the ability to perform a task safely and effectively [10], while proficiency often implies a higher level of performance consistency over time. Credentialing, in this context, is the formal process by which an institution verifies and authorises a surgeon’s qualifications to perform robotic procedures independently [11]. It typically represents the endpoint of supervised training and the entry into independent practice, though the specific threshold for credentialing can vary significantly between institutions and between residents and established consultants/attending surgeons. Currently, no universally accepted credentialing standards exist for robotic general surgery, leading to marked heterogeneity in surgical quality and practice. The current industry-driven model introduces potential conflicts of interest due to reliance on vendor-specific credentialing, often without independent oversight. The establishment of independent credentialing bodies could ensure transparency and neutrality, thus enhancing trust, standardisation, and fairness within robotic surgical training.

**Table 1:** Definitions in the context of robotic surgical training

Term	Definition
Credentialing	The formal process by which an institution verifies a surgeon’s qualifications to perform robotic procedures independently
Privileging	The institutional authorisation to perform specific robotic procedures
Competency	A surgeon’s ability to perform a task safely and efficiently, to an accepted minimum standard
Proficiency	A surgeon’s high level of consistent performance over time and is established by passing benchmarking
Benchmarking	The use of objective performance metrics or other standardised assessment, derived from expert populations, to define minimum competency and proficiency targets

The need for structured, standardised, and scalable training and credentialing frameworks is critical to ensure high-quality clinical standards and patient safety through the introduction and application of key training standards [12, 13]. We explore the current challenges and future directions for robotic surgical training in general surgery and propose a credentialing pathway aimed at addressing these barriers through competency-based progression and platform-agnostic standards.

## Existing robotic training frameworks

Robotic surgical education has evolved substantially over the past decade; however, initiatives remain fragmented in scope and implementation, and no unified, cross-subspeciality credentialing framework currently exists for robotic general surgery. There are a number of curricula published for training in robotic surgery.

The Fundamentals of Robotic Surgery (FRS) curriculum represents an established multi-speciality, proficiency-based training programme for foundational robotic skills. It incorporates simulation-based training, cognitive modules, and objective performance assessment, with evidence supporting construct and content validity. FRS provides a transferable foundation in console control, camera navigation, clutching, and instrument manipulation independent of specific procedures or specialities [14]. The Royal College of Surgeons in Ireland (RCSI) and the International Medical Robotics Academy (IMRA) have recently published platform-agnostic curricula [15–17].

Speciality-specific pathways have also been developed. The European Association of Robotic Urology Surgeons (ERUS) introduced a modular curriculum integrating simulation, stepwise procedural progression, and validated assessment, with prospective clinical validation [18]. Expert consensus recommendations for robotic credentialing, including those led by Stefanidis and colleagues, have defined structured requirements for simulation exposure, proctorship, and case documentation [12] and the Robotic Surgery Education Working Group proposed recommendations for integrating robotic training into residency, including defined bedside and early-console benchmarks [19].

Objective assessment tools are increasingly available. The Global Evaluative Assessment of Robotic Skills (GEARS) remains the most widely validated instrument for structured technical performance assessment [20, 21]. Video-based blinded review and automated console-derived performance metrics are emerging as complementary methods to support competency-based progression [22, 23].

Despite these advances, current frameworks share four limitations. First, most are speciality-specific or geographically limited. Second, many focus on discrete components of training, simulation, procedural modules, rather than a continuous pathway from foundational skills through independent practice and revalidation. Third, vendor-led certification continues to dominate early robotic exposure in many institutions, especially in senior surgeons, introducing variability and potential conflicts of interest. Finally, they do not differentiate between surgeons of variable experience, including early-years residents and experienced robotic-naive attending surgeons.

While validated components of robotic training exist, they have not been integrated into a unified, operational credentialing architecture applicable across platforms, institutions, and career stages within general surgery. Previous publications have endorsed components of robotic training pathways. The framework proposed in this manuscript seeks to consolidate existing validated elements into a structured, competency-based, and platform-agnostic pathway.

## The need for standardised robotic training and credentialing

Robotic surgery represents a fundamentally different technological transition from previous shifts such as the adoption of laparoscopy. Earlier innovations modified surgical access while preserving the direct relationship between surgeon, instruments, and patient but robotic surgery replaces this relationship with a digitally mediated interface, introduces platform-dependent operating environments, and enables continuous objective performance measurement [24]. These differences alter how surgical skill is acquired, assessed, and maintained and therefore require a more deliberate and standardised approach to credentialing than previous surgical transitions. There will be differences in how training is delivered locally, but harmonisation of credentialing would allow better portability and flexibility of training.

Robotic surgery separates the surgeon physically from the patient, reducing direct haptic feedback and requiring reliance on visual and instrument-derived cues to judge tissue interaction and force application. At the same time, surgeons must master a complex technological interface alongside procedural technique, including console ergonomics, camera control, clutching, articulated instrumentation, docking, and troubleshooting. As multiple robotic platforms emerge, variation in interface design further complicates skill transfer and reinforces the need for structured foundational training combined with platform-specific credentialing.

The robotic operating environment also reconfigures team dynamics. The console surgeon is physically removed from the bedside, altering communication, situational awareness, and emergency response processes. Safe robotic practice therefore depends not only on technical console proficiency but also on clearly defined team roles, communication strategies, and non-technical skills that differ from those required in open or laparoscopic surgery [25].

Importantly, robotic systems generate detailed performance data, including instrument motion metrics and console analytics. This new capacity for objective measurement distinguishes robotics from previous surgical innovations and provides an opportunity to move beyond volume-based training models towards competency-based credentialing supported by measurable performance standards [26].

Taking these unique features of robotic general surgery together, a clearly defined, competency-based credentialing framework integrating simulation, objective assessment, modular procedural progression, and ongoing revalidation is therefore essential to ensure safe and consistent adoption across institutions and career stages.

## Robotic training challenges and barriers

Despite the growing integration of robotic platforms into general surgical practice, training pathways have failed to evolve in parallel. The rapid expansion of robotic surgery has outpaced the development of structured educational frameworks, leading to issues regarding variable robotic access, pedagogical, and logistical challenges.

### Access to robotic platforms

One of the key immediate challenges to robotic surgical training is limited access to robotic systems. Given financial costs of acquiring a robotic system, many institutions have developed multi-speciality robotic programmes to ensure effective clinical volume, offset costs and maximise utilisation [27]. However, this approach limits the broader opportunities for hands-on training for junior and senior surgeons within their individual specialities, leading to a delay in exposure until existing teams’ learning curves are overcome. Furthermore, geographic variation in robotic infrastructure further exacerbates inequalities in robotic access for surgeons and patients alike [28, 29]. The uneven distribution of robotic surgery and training across the workforce reinforces the notion of a ‘postcode lottery’ for surgical innovation and patient access [30]. These challenges become even more pronounced in low-income and middle-income countries [31, 32].

Furthermore, in many healthcare systems such as the NHS in the UK, early-years residents will rotate between departments and institutions, which may have different robotic platforms. A platform-agnostic common basic skills stem within a credentialing framework will promote skill transference and progression despite this challenge and allow for future surgeons to operate across multiple platforms if needed.

### System availability

At an institutional level, when a new system is adopted or a surgeon wants to begin operative practice, the availability and rationing of case-volume for an individual may be a challenge. In developing credentialing models, consideration should be given to whether a hospital operates a *single-system, speciality-based* programme or a broader *multi-speciality* arrangement within a teaching or private hospital setting. Early-years practice may focus on establishing familiarity with already well-defined procedures, while later years can introduce more complex work. A balance is required between *support* and *checking*, framed within a clear *legal and governance structure*.

Case volumes should guide system utilisation. Using an illustrative example from a theoretical unit suggested by the author’s experience, a single system performing 375 cases/year under a standard 9–5, 5-day week equates to 1.5 cases/day; inclusion of weekends raises this to 425 cases/year and extended hours to 500 cases/year. To achieve one case per week per surgeon, 10 surgeons could develop expertise; with two surgeons sharing a list, a maximum of 20 surgeons can be supported. This may translate to multi-speciality allocation and if demand exceeds capacity, an additional robotic system should be considered.

### Lack of standardisation

There is currently no universally accepted curriculum or training pathway for robotic general surgery, due in part to the breadth of the speciality and geographical differences in practice [7]. Across the world, institutions differ in how they deliver and assess training, with no consistent benchmarks for competency or proficiency. The absence of standardised curricula, milestones, and credentialing requirements makes it difficult to compare training outcomes across hospitals, regions, and countries. Moreover, feedback mechanisms are highly variable, with some trainees receiving structured assessment while others rely on informal feedback during cases [33]. This heterogeneity ultimately undermines the ability to safely evaluate performance and outcomes in robotic surgery. Previous Delphi consensus has highlighted common core components of robotic training pathways [34].

### Parallel learning curves

A unique challenge in robotic surgery is the phenomenon of parallel learning curves, whereby surgeons and theatre teams are learning the platform simultaneously. This dual learning dynamic can strain educational environments, particularly for surgical trainees. During the learning curve phase, senior surgeons are less likely to be able to provide structured mentorship to trainees, whilst building their own technical experience [29]. This can lead to delayed training opportunities and skills acquisition for surgical trainees, due to a loss of training opportunities or early adoption of innovative training opportunities. Developing innovative training opportunities, including parallel component training and sequentially training surgical trainees, is a safe and effective manner of delivering high-quality robotic training [35]. Mentorship models need to develop to support robotic training, including reverse mentorship, with younger surgeons developing robotic practices and supporting senior colleagues through peer-to-peer knowledge transfer and support.

### Complexity of robotic systems

Robotic surgery is associated with significant cognitive and technical load [36]. Unlike traditional open or laparoscopic approaches, it requires surgeons to master console-based control systems, spatially decontextualised hand–eye coordination, multi-step docking, troubleshooting, and team training. Robotic-assisted general surgery is unique for several reasons: There is a physical separation from the surgeon and the patient, with reduced haptic feedback, the technological interface requires mastery alongside developing manual surgical skills, and reconfiguration of the traditional surgical team is required to enable docking, bedside assistance and troubleshooting of operation and system-related issues. Mastery of these skills requires deliberate, structured practice, a demand that can often be unmet in current training environments.

### Multiple robotic platforms

The surgical robotics market is rapidly diversifying. While the Da Vinci Surgical System (Intuitive Surgical, Sunnyvale CA) remains the dominant platform, newer systems such as Versius (CMR Surgical, Cambridge UK), HUGO (Medtronic, Dublin, Ireland), and Senhance (Asensus Surgical) are now available in many jurisdictions. Each platform has its own proprietary instrumentation, user interface, and learning curve. There is little data on platform-to-platform interactions with regard to learning curve and transference of generic robotic skills. Initial evidence suggests that overall robotic platform and procedural experience can reduce the learning curve when adopting new robotic systems [37]. This requires further investigation particularly for rotating surgical trainees, who are likely to have the most exposure to multiple platforms throughout training. There is currently no single platform-agnostic early training model to accelerate surgical and platform-based training leading to a robotic credential. Whilst platform-specific features in the interface and technology may contribute to this training challenge, there are common robotic principles. A standardised credentialing process that includes progression through a platform-agnostic core skills programme, followed by platform-specific and speciality-specific procedural skills programme, will help to minimise this problem whilst maintaining high quality and standardised training. The inclusion of parallel industry-led device training, in which surgeons are assessed on the principles and technology of a specific robotic device, will ensure that surgeons do not have superficial training on multiple platforms.

Moreover, the adoption of multiple robotic platforms raises significant regulatory and liability considerations. Credentialing surgeons across different systems demands clear regulatory oversight, particularly concerning responsibilities in the event of adverse outcomes or technical complications. Establishing a robust governance framework could mitigate risks and clarify institutional and surgeon liabilities associated with cross-platform credentialing.

### Time and service pressures

The demands of clinical service often limit the time available for formal surgical training [38]. Robotic cases are typically longer in duration than laparoscopic procedures [39], sometimes making it difficult to justify trainee involvement in high-volume settings. This is particularly acute in systems under strain, where surgical efficiency and theatre throughput are prioritised. Without protected time for learning, robotic training is often ad hoc and opportunistic, rather than structured and goal-directed [40]. Ensuring trainees are ‘robot ready’ to take advantage of these ad hoc training opportunities when they arise is important. The recent RACS working party report endorses early integration of robotic surgery in surgical registrar training [41].

### Learner support and feedback

There is a notable deficit in structured learner support and feedback in many robotic training environments. Access to skilled mentors varies considerably, and the use of video-based feedback, which has been shown to accelerate skill acquisition [23], is still uncommon. Similarly, objective performance tracking and benchmarking tools are rarely employed. This lack of feedback infrastructure prevents robust evaluation and hampers learners’ ability to identify strengths and address areas for improvement, slowing the path to proficiency. The ability of a robotic system to record operative metrics could be transformative to the future of surgical training [42, 43].

## Solutions

Addressing the challenges in robotic surgical training requires a multifaceted approach that incorporates educational innovation, system-level support, and thoughtful integration of credentialing frameworks. Many of the barriers identified, such as limited access, lack of standardisation, and parallel learning curves, are embedded in existing institutional and cultural structures. However, emerging tools, practices, and technologies, including simulation, extended-reality platforms, and video-based assessments, offer promising avenues for scalable and equitable training. Importantly, solutions must be tailored to the differing needs of junior trainees and senior surgeons, recognising their distinct learning trajectories and professional responsibilities.

Table 2 outlines proposed interventions mapped to each key challenge. These solutions are designed to enhance both individual learning and systemic capacity, forming the basis for a robust and standardised credentialing pathway.

**Table 2:** Challenges and solutions to robotic training in general surgery

Challenge	Solution	Description and implementation
Access to robotic platforms	Simulation-based training	The use of simulation methods, including improved extended reality, dry lab and wet lab models can help individuals progress up their early learning curve without the need for in-theatre practice. Integrating access and standardised simulation training into training programmes can improve efficiency of training, reducing reliance on theatre access
	Extended reality (XR) platforms	Use XR to scale training where physical system time is limited: cognitive rehearsal, procedural orientation, and team-based scenarios (e.g. docking failures/emergency undocking). XR supports deliberate practice outside theatres and can standardise exposure across sites with variable robotic capacity
	Observerships and modular access programmes	Create regional hub-and-spoke observerships and modular ‘access blocks’ (structured exposure weeks) across institutions to address geographic inequity and accelerate early adoption. This is particularly relevant where robotic distribution is uneven and countries where robotic surgery is being established
	Platform-agnostic basic skills training	In some situations, residents may rotate between departments and institutions in their early learning curve. A platform-agnostic common stem for basic skills will allow for continued progression and for surgeons to develop proficiency on multiple platforms in the future
System availability	Formal governance structure within institutions	Each institution should establish a robotic governance committee, including clinicians, that includes a focus on training + credentialing, to define access rules, progression criteria, proctor requirements, and escalation pathways for adverse events. Align governance with published training consensuses and national recommendations
	Structured rationing of individual platforms between surgeons and specialities	Implement transparent allocation of lists by speciality and surgeon stage (training vs service), including protected training lists and staged autonomy. Multi-speciality programmes should explicitly reserve capacity for training rather than defaulting to service efficiency
Lack of standardisation	National or institutional curriculum frameworks	Adopt an agreed curriculum architecture for training residents integrated into their overall training curricula, led by a relevant national institution (e.g. Royal College of Surgery, Surgical Speciality Association)
	Defined milestones and competency metrics	Specify milestone gates using validated tools and measurable endpoints (e.g. GEARS for technical performance; structured video review; console-derived metrics). GEARS is widely validated and can support standardised progression decisions across settings {citation}
	Credentialing schemes aligned to training stages	Define stage-specific outcomes (e.g. ‘robot-ready’ during residency, developed basic skills and undertaking procedural component training; ‘robot-competent’ for supervised procedural delivery; ‘robot-proficient’ for independent practice) and align credentialing gates to these stages. National recommendations support earlier integration into training when milestones are clearly defined Training should be tailored depending on whether the surgeon is a resident or experienced senior consultant/attending. However, the benchmarks and credentialing framework stages and components should remain consistent
Parallel learning curves	Component-based training pathways	Adopt modular participation models (e.g. bedside + discrete console components) to enable trainee progression while supervising surgeons are still developing proficiency
	Reverse mentorship models	Formalise peer/near-peer teaching where early adopters and trainees support senior surgeons’ platform familiarity and workflow optimisation while maintaining governance and patient safety oversight. This addresses capacity constraints during early programme establishment
Complexity of robotic systems	Stepwise simulation and cognitive task training	Front-load training in console interface skills, troubleshooting, ergonomics, and cognitive workload management before live operating. This addresses robotics’ distinct cognitive ergonomics and interface-mediated demands
	Scenario-based simulation training	Use scenario simulation for low-frequency, high-risk events (system failure, undocking, bleeding at console separation) to build team readiness and reduce safety risk during early adoption
	Deliberate practice with feedback	Implement scheduled deliberate practice with structured feedback loops, using objective measures and coaching. Feedback infrastructure (including video review) accelerates acquisition and reduces variability in informal case-by-case teaching
	Use of advanced models for simulated practice	Use wet lab, bio tissue, hydrogel, or cadaveric models for procedural steps where simulation fidelity is required depending on local availability. Combine with deconstructed procedural descriptions to structure modular learning and assessment
Time and service pressure	Protected robotic training time	Include protected robotic training time in job plans and programme timetables; without this, training becomes ad hoc and inequitable. Service pressure and burnout are recognised barriers to consistent training delivery
	Modular training integrated into service provision	Embed training into routine lists using staged, component-based autonomy (e.g. defined steps per case) to balance throughput and development. Modular deconstruction supports efficient progression without prolonging cases excessively
Learner support and feedback	Structured mentorship and coaching	Create formal mentorship and proctoring roles with clear criteria for progression and sign-off, aligned to credentialing consensus recommendations. Mentorship should include both technical and team/non-technical domains
	Video-based assessment and self-reflection	Adopt routine video capture with structured review (self, mentor, and where possible blinded/peer review) to standardise feedback and accelerate skill development. Video-based assessment can be used for key index procedures, improving efficiency of learning
	Real-time performance tracking with objective metrics	Adopt routine video capture with structured review (self, mentor, and where possible blinded/peer review) to standardise feedback and accelerate skill development

## Proposed credentialing pathway

Drawing on the published literature reviewed above, existing consensus frameworks, and the authors’ collective expertise in robotic surgical training across five countries, we propose a credentialing pathway, standardised as a basic model between countries, general surgical specialities and that can be adapted for both training residents and experienced consultant/attending-level surgeons.

During residency, previous literature has supported the inclusion of standardised robotic training early in their programme, and the NHS robotic surgery implementation guidelines advocate for the introduction of robotic principles as early as medical school. With this in consideration, training should start early and this multi-phase model can take residents through three distinct phases: [1] ‘robot-ready’ in early residency, where trainees complete basic skills training and undertake component-based procedural training under supervision, [2] robot-competent, in late residency or fellowship as they complete procedural skills training able to complete procedures under supervision and meet competency assessment benchmarks, and [3] ‘robot proficient’ where surgeons have achieved credentialing and have consistent independent operative performance.

A platform-agnostic foundational phase ensures early exposure to robotic principles through simulation, observership, and standardised curricula [44]. This could mitigate disparities in access and prepare trainees for multiple systems. Subsequent speciality-specific procedural training incorporates bedside experience, modular case participation, and supervised progression, consistenfit with task deconstruction models [25].

A competency-based progression model enables individualised advancement based on performance, supported by objective assessment tools including operative metrics, structured feedback, and video-based evaluation. Non-technical skills training (e.g. NOTSS) is integrated in parallel, reflecting their recognised role in surgical safety [25], particularly important in robotic surgery given the additional complexities. Due to the impact on the whole team, individuals completing the credentialing pathway should attend robotic-specific NOTSS training [45] as individuals and part of the wider team.

**Fig. 1:**
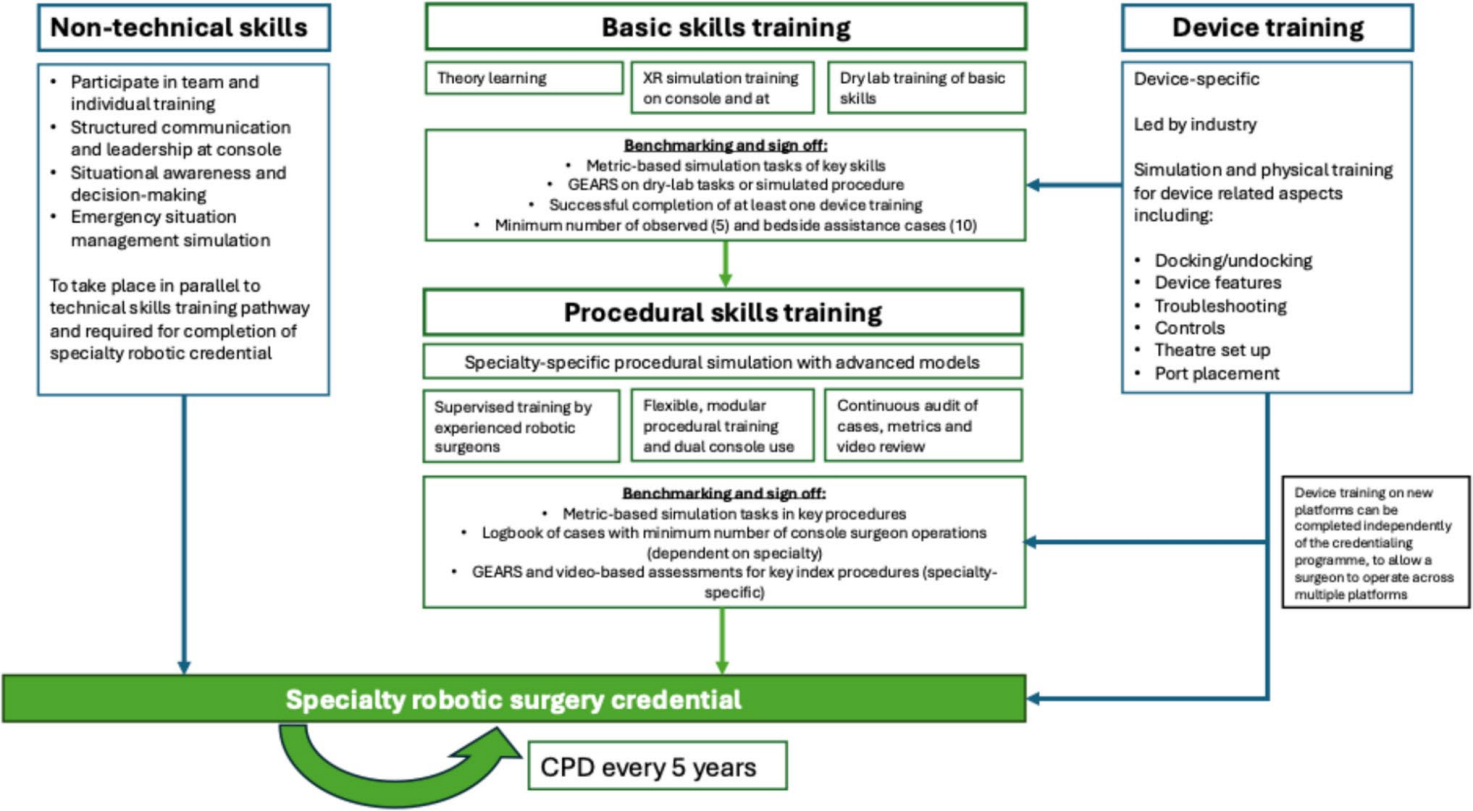
Proposed credentialing pathway

Credentialing is granted upon demonstration of defined competencies across technical and non-technical domains, with revalidation mechanisms to ensure ongoing proficiency. The pathway is adaptable to varying institutional resources and accommodates both trainee and consultant learners. Figure 1 outlines a suggested pathway for a surgeon to achieve a robotic credential, with standardised assessment required to pass each stage of device, core, and procedural training. The contents of each stage may be variable between individual surgeons and institutes and flexible in their order. For example, a training surgeon may undertake wet-lab training with hydrogel models alongside part-procedural console training to improve their learning curve progress. A surgeon with established independent practice may require less time in part-procedural training due to their established procedural skills; however, standardised assessment prior to credentialing will continue to ensure high-quality robotic surgical practice across platforms, institutions and specialities.

It is recognised that credentialing requirements can differ significantly between institutions. Common components often include industry certification, trainer competency verification, operative logbook review, and completion of in-service and online educational modules. Standardising these components through a national or international framework would help mitigate variability and ensure consistent competency benchmarks. Proposed assessments at each stage are outlined in Table 3. Thresholds for specific assessments and benchmarking and absolute case numbers may vary between specialities based on the complexity of index procedures and a framework that allows for validated and consensus-driven changes in these numbers may be the most achievable method to drive high-quality standardisation in assessment.

**Table 3:** Assessment required to pass each stage of the credentialing process

Stage	Assessments
Device training	• Completion of device/manufacturer training and certification • Knowledge-based assessment of system components, safety, and troubleshooting • Demonstration of safe system setup, docking, and emergency undocking • Basic console familiarisation on simulator or dry-lab platform, passing metric-based assessment
Basic skills training	• Simulation-based proficiency benchmarks using validated metrics set by experts and consensus including, but not limited to camera control, clutching, instrument manipulation, energy use • Standardised assessment of dry lab or simulation • Faculty/mentor sign-off for progression to supervised procedural training • Minimum number of live cases as bedside assist: 10 (19) • Completion of at least 1 certified device training
Procedural training	• Completion of a speciality-specific procedural skills simulation course with standardised assessment of skills on advanced models (using GEARS, OSATS, or video-based assessment) • Logbook of supervised cases with defined procedural component progression, numbers and index cases variable between specialities as appropriate (18) • Structured intraoperative assessment by proctor/trainer (e.g. GEARS, procedure-specific assessment, or video review) • Demonstration of safe independent completion of key procedural steps • Assessment of decision-making, troubleshooting, and team communication as completion of non-technical skills training course • Proctor/trainer sign-off for independent practice/credentialing based on all of the above
Continuing professional development	• Ongoing operative volume and outcome monitoring • Periodic peer or video-based review of selected cases • Evidence of continued robotic CPD (simulation, courses, or team training) • Institutional review aligned with local governance requirements • Important principle but any requirements should be individualised based on speciality, access, and local case-mix

## Continuing professional development

Continuing professional development should form an integral component of robotic credentialing frameworks to ensure ongoing proficiency following initial accreditation. Institutions and credentialing bodies should include periodic review of operative case volume, outcomes, and complication profiles, supported by prior consensus [12], alongside structured peer or video-based assessment where feasible. Continued engagement with robotic practice, simulation-based refresher training and multidisciplinary team training should be encouraged to maintain both technical and non-technical skills. As robotic platforms and procedural applications evolve, revalidation processes will require iterative refinement to ensure sustained competency, patient safety, and consistency of practice across institutions. This aspect should be broad as case-mix and access may be different across specialities and institutions and therefore may be individualised.

## Implementation of a national or international credentialing framework

Operationalising a standardised credentialing framework will require coordinated action at institutional, national, and international levels. At an institutional level, formal robotic governance structures are needed to oversee access, progression, and credentialing processes, ensuring transparency and alignment between service delivery and training priorities; this should be recommended or mandated from a national policy level.

Second, integration of early simulation-based foundational training and structured assessment into existing surgical curricula will be essential to ensure equitable early exposure for trainees and structured transition pathways for consultant/attending surgeons adopting robotic techniques. At a national and professional level, surgical organisations and training bodies will play a critical role in endorsing core curricula, defining competency benchmarks, and supporting independent credentialing processes. Alignment with existing validated programmes and consensus recommendations will facilitate portability of training and reduce reliance on vendor-specific certification pathways. Regional training hubs and shared simulation resources may further support scalable implementation, particularly in settings with limited robotic infrastructure.

Credentialing frameworks must also remain adaptable to differing healthcare environments while maintaining consistent core standards. Prospective evaluation and iterative refinement will be required to ensure that training pathways translate into improved technical performance, patient safety, and equitable access to robotic surgery. Establishing mechanisms for ongoing review and revalidation will be central to sustaining proficiency as robotic technologies and surgical applications continue to evolve.

It should also be noted that the development of a validated framework, including assessment of metrics and revalidation structures, will take time, money, and leadership. Collaboration between industry, international institutions, and funding bodies can help to create a single common and standardised pathway. In the meantime, efforts should be focussed on the delivery of safe training and improving access in the short term.

## Conclusion

The rapid adoption of robotic surgery within general surgical practice presents both an opportunity and a challenge. While the benefits of robotic platforms in terms of precision, ergonomics, and outcomes are increasingly evident, the infrastructure for safe and effective training has not kept pace. Current pathways are hindered by variable access, lack of standardisation, and parallel learning curves, particularly as both junior and senior surgeons seek to upskill simultaneously.

Credentialing offers a potential solution to many of these challenges. A structured, competency-based, and platform-agnostic credentialing pathway can provide clarity, ensure patient safety, and support equitable access to training across institutions. By incorporating simulation, modular procedural exposure, non-technical skills development, and ongoing assessment, such a framework can help define standards of practice and build confidence in robotic proficiency across all career stages. Given the variability in institutional practices, credentialing frameworks must be flexible yet standardised, accommodating differences in resources, regulatory environments, and institutional policies.

To make this vision a reality, coordinated action is required from professional bodies, training institutions, and healthcare systems. Stakeholders must invest in simulation infrastructure, endorse standardised curricula, and commit to consistent assessment and revalidation processes. The future of robotic surgery will not be defined by technology alone, but by the ability of our systems to train, credential, and support the surgeons who use it.

## Data Availability

No new data was presented in this manuscript.
